# Delamination Study in Edge Trimming of Basalt Fiber Reinforced Plastics (BFRP)

**DOI:** 10.3390/ma11081418

**Published:** 2018-08-13

**Authors:** Maria Dolores Navarro-Mas, Juan Antonio García-Manrique, Maria Desamparados Meseguer, Isabel Ordeig, Ana Isabel Sánchez

**Affiliations:** 1 Department of Mechanical Engineering and Materials, Universitat Politècnica de València, Camino de Vera s/n 46022 Valencia, 46022 València, Spain; jugarcia@mcm.upv.es (J.A.G.-M.); amesegue@mcm.upv.es (M.D.M.); iordeig@mcm.upv.es (I.O.); 2 Department of Statistics and Operational Research, Universitat Politècnica de València, Camino de Vera s/n 46022 Valencia, 46022 València, Spain; aisanche@eio.upv.es

**Keywords:** basalt fiber reinforced plastic (BFRP), delamination, edge trimming, tool wear

## Abstract

Although there are many machining studies of carbon and glass fiber reinforced plastics, delamination and tool wear of basalt fiber reinforced plastics (BFRP) in edge trimming has not yet studied. This paper presents an end milling study of BFRP fabricated by resin transfer molding (RTM), to evaluate delamination types at the top layer of the machined edge with different cutting conditions (cutting speed, feed rate and depth of cut) and fiber volume fraction (40% and 60%). This work quantifies delamination types, using a parameter Sd/L, that evaluates the delamination area (Sd) and the length (L), taking into account tool position in the yarn and movement of yarns during RTM process, which show the random nature of delamination. Delamination was present in all materials with 60% of fiber volume. High values of tool wear did not permit to machine the material due to an excessive delamination. Type II delamination was the most usual delamination type and depth of cut has influence on this type of delamination.

## 1. Introduction

Nowadays, aeronautical and automotive manufacturers use fiber reinforced composites, because of their properties of light weight and high strength [1]. In many cases, these composites are manufactured using processes such as resin transfer molding (RTM), vacuum infusion, prepegs, etc. Nevertheless, a machining process step is necessary after curing to meet the required tolerances and final shape of the part [2] or to allow the union of different components. Edge trimming and drilling are the most extended machining processes [3].

Machining damage is unavoidable due to the high mechanical resistance of the fibers, which causes excessive tool wear and poor surface finish [4]. In edge trimming, delamination of the top and/or the bottom layer of the composite, due to the axial tool force generated by the helix inclination angle of the milling tool is another important factor that affects dimensional precision and mechanical performance of parts. Delamination occurs because the fiber is not restrained by the outside in the top layer, and the tool bends the fibers outward and deflect them away from the plane of the laminate. Types of delamination can be classified into three groups (I, II and III) [5]. Type I delamination characterizes areas where the surface fibers have been broken some distance inward from the trimmed edge. Type II delamination takes into account uncut fibers that protrude from the trimmed edge. Type I/II is a combination of the two previous types. Type III delamination describes fibers parallel to the machining surface.

Studies on delamination are focused on drilling and milling (slot milling and edge trimming). Main factors that affect delamination are the tool wear, determined by the machining conditions, tool geometry, and volume and orientation of fiber in the composite material [6,7].

In drilling, a study based on digital image analysis defines different factors to evaluate delamination [8]. Firstly, delamination factor (F_d_ = D_max_/D_o_) is calculated as the ratio of the maximum diameter of the delamination zone (D_max_) to the drill diameter (D_o_). Secondly, the adjusted delamination factor (F_d_) represents the conventional delamination factor (F_d_) plus the damage area contribution. Some authors have used this delamination factor to evaluate spindle speed, feed rate and the point angle in delamination of carbon fiber reinforced plastics (CFRP) with response surface methodology [9] generating a second order regression model. With high spindle speed and low feed rate, delamination reduces at the entrance of the hole.

In milling works, there are mainly two different processes studied: slot milling and edge trimming. In slot milling, recent studies evaluate the occurrence and propagation of delamination in CFRP [10] concluding that delamination is influenced by fiber orientation and tool wear. On the other hand, the undulation of the woven yarn provokes different types of delamination: fiber protrusion and surface damage [11]. Cutting conditions (spindle speed, feed rate and depth of cut) and number of end milling flutes are evaluated to minimize delamination (delamination factor F_d_) and surface roughness using neural network and genetic algorithm in glass fiber reinforced plastics (GFRP). As conclusion, F_d_ increases with feed rate and decreases with spindle speed [12].

In edge trimming, surface delamination is random [13]. Damage caused by delamination in edge trimming of CFRP using burr tools can be quantified in terms of delamination depth and frequency of occurrence per length, taking into account feed rate, cutting speed and tool wear [13]. Delamination increases when feed rate and machining length increase and the spindle speed decrease [14].

Most studies that approach fiber reinforced composites machining are focalized in glass and carbon fibers, however, there are almost no studies with basalt fiber reinforced plastic (BFRP) [15]. Basalt is a natural mineral with good properties, with a similar mechanical behavior that carbon fiber [16]. BFRP process manufacturing is similar to GFRP, without additives and with a lower amount of energy [17,18].

In this paper, a plain weave basalt fiber reinforced plastic is milling with edge trimming in order to study delamination. Cutting conditions (cutting speed, feed per tooth and depth of cut) and fiber volume fraction are varying, as tool wear is evaluated. Tool selection takes into account a commercial tool suitable for edge trimming, with a high productivity due to a long tool life for chosen cutting parameters and with a high cutting length. This work studies and quantifies delamination types depending on the position that the tool hits the yarn, and stablishes parameters to compare different experiments.

## 2. Materials and Methods

**Figure 1:**
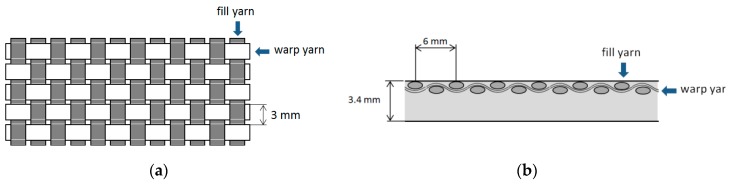
(**a**) Bidirectional panel; (**b**) Top layer laminate.

**Figure 2:**
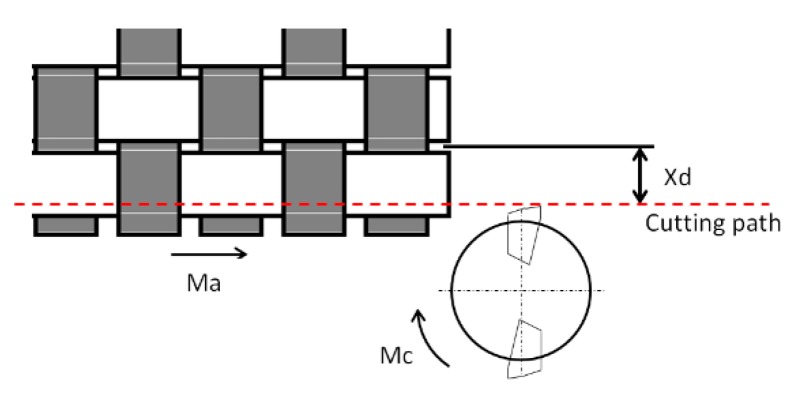
Xd Schema.

Basalt fiber reinforce plastic (BFRP) laminates used in this study were manufactured using RTM process. The material was a rectangular (420 mm × 260 mm) bidirectional panel with plies of plain weave basalt fibers impregnated with an epoxy resin (Prime 20 LV Gurit, Switzerland). Fiber orientation was 90°. Figure 1 shows the laminate and its top layer. In the top layer, it is possible to see different zones, depending on the quantity of resin and the position of the fibers. The thickness of the material was 3.4 mm. Step between yarns was 3 mm, which represents a limit for Xd (Figure 2), Xd being the distance of the warp yarn from the trimmed edge until the next dip below the crossing fill yarn [11].

Edge trimming operation (down milling) was conducted using a Kondia B-500 milling machine (Kondia, Elgoibar, Spain), with a spindle power of 6 KW and a maximum rotation speed of 6000 rpm. A Mitsubishi Materials milling tool holder of diameter 25 mm (AXD4000R252SA25SA Mitsubishi Materials, Japan), with two exchangeable uncoated carbide cutting inserts (XDGX175008PDFR-GL TF15 Mitsubishi Materials, Japan) was used for machining.

**Figure 3:**
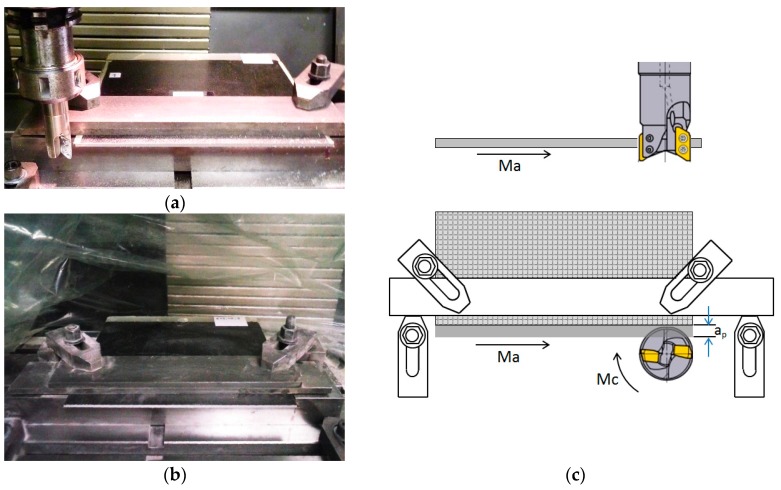
(**a**) Fixture with clamps; (**b**) Film bag; (**c**) Down milling schema.

Clamps were used in the milling machine to avoid vibrations and displacement of the laminate, leaving the minimum cantilevered possible. A film bag involving machining zone was used to avoid dust (Figure 3).

Cutting conditions (cutting speed, feed per tooth and depth of cut) and material characteristics (fiber volume fraction) were considered to study delamination, taking into account the flank wear of the tool. The Taguchi’s method for four variables at two-levels was used to elaborate the design of experiments. Table 1 shows the variables studied and their levels.

In this paper, a L_8_ orthogonal array was selected for determining combinations of factor levels to use for each experimental case. Table 2 shows the values of the parameters for each experiment using an L_8_ orthogonal array. Due to the time consumed for each experiment, a full factorial design was not used, in order to reduce the number of experiments. No replicas were done, because of the random nature of delamination.

In every experiment, a tool with new inserts machined without coolant for 80 min. Different stopping times (30, 55, and 80 min), or cutting times (Tc), were made to measure delamination and flank wear (Vb) in both cutting inserts. Measuring of delamination and flank wear are carried out recording and analyzing images of the laminate material and the clearance face of the tool, respectively. An Olympus SZ61 microscope (Olympus, Japan) and image processing software (Olympus stream software, GIMP 2.8) with pixel calibration was used to measure delamination and flank wear. In flank wear, measurements were repeated two times in every insert and a maximum value was chosen for every experiment.

## 3. Results and Discussion

### 3.1. Types of Delamination

**Figure 4:**
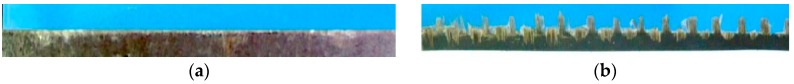
(**a**) Fv 40% without delamination; (**b**) Fv 60% with high delamination.

In the experimental results obtained in this study, laminates with a 40% of fiber in volume do not present delamination (Figure 4a), because the resin restrains the fiber in the top layer. Laminate with a 60% of fiber in volume presents delamination (Figure 4b) at the top surface.

**Figure 5:**
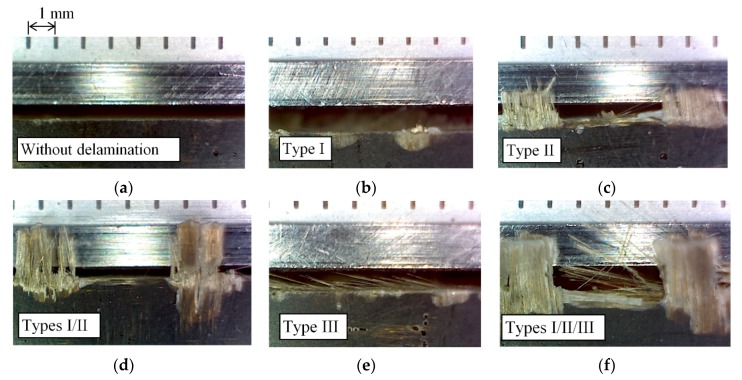
(**a**) Top layer without delamination (Fv 40%); (**b**) Surface damage (Type I); (**c**) Fiber protrusion (Type II); (**d**) Types I and II delamination; (**e**) Type III delamination; (**f**) All delamination Types.

Delamination can be classified in three Types (I, II and III) [5], as shown in Figure 5.

**Figure 6:**
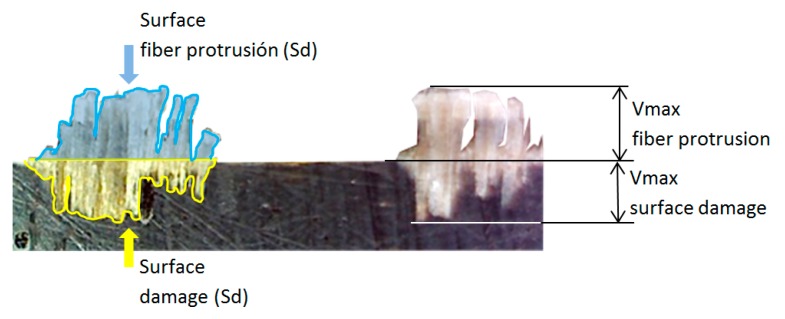
Surface damage and fiber protrusion measurement.

For each delamination type, the maximum value (Vmax) and the surface occupied (Sd) were measured (Figure 6). Only Vmax has been measured by some authors [11,13], but not Sd (surface damage or surface protrusion).

Table 3 shows values of tool wear and surface damage (Type I) for each stop. Table 4 shows values of tool wear and fiber protrusion (Types II and III) for each stop. A length of 80 mm was measured and the value of “% L” represents the percentage of this length with delamination. In most experiments “% L” occupied for delamination is important, but values of Sd are maintained low, so they are acceptable. Values for Sd and Vmax were expected to increase as cutting time and tool wear increase as well. Nevertheless, some values decrease instead of increase. It is due to the random nature of delamination and the tool position when it hits the yarn. This will be explained in more detail in Section 3.3.

In Experiment 2, cutting conditions were the worst (high cutting speed, high feed rate and high depth of cut) and flank wear increased very highly and no cutting was possible after 30 min. This experiment only shows values at 5, 10 and 30 min. High tool wear depends on high cutting speed and high feed per tooth [13].

**Figure 7:**
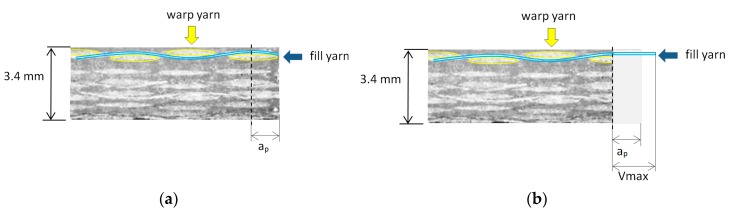
(**a**) Depth of cut (ap) before machining; (**b**) Vmax and ap after machining.

In Type I delamination, Vmax value is less than yarn size, and the highest values are with large feed per tooth. In Type II delamination, in most cases, Vmax values are larger than depth of cut (ap). This can be explained because, originally, the fill yarn has a sine wave form and after cutting the yarn is stretched (Figure 7), according to other studies [14].

When ap is less than yarn size, Type I delamination can be larger than Type II.

In Experiment 3 at 80 min and in Experiment 4 at 30 min, values of Vmax were very high because Type II and III delamination were superimposed. In these experiments, Types I and III were lower than Type II, being Type II the most critical delamination.

### 3.2. Relation between Fiber Orientation and Fiber Position in Laminate Respect to Machining Direction

**Figure 8:**
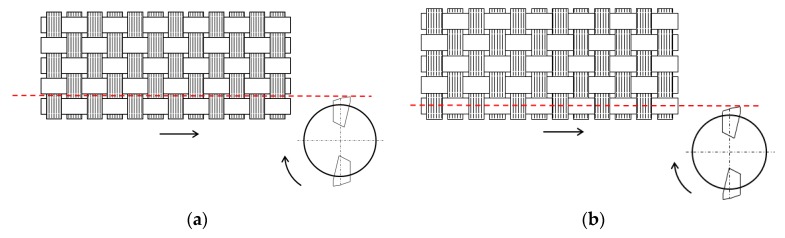
(**a**) Low uniform delamination; (**b**) High uniform delamination.

Delamination measurement is needed to justify the phenomenon that occurs when machining a plain weave sample. Relation between fiber orientation and position in the laminate to machining direction is an important parameter. Position fiber refers to the point where tool hits the width of the yarn. Two different kinds of delamination can be found: uniform and sine wave form. On the trimmed edge, delamination is uniform if the machining direction is aligned with fiber orientation. Delamination can be low or high, depending on the position the tool hits the yarn (Figure 8). Low delamination occurs when tool trajectory is into the space between yarns, while higher delamination occurs if the tool trajectory passes in the center of the yarn.

**Figure 9:**
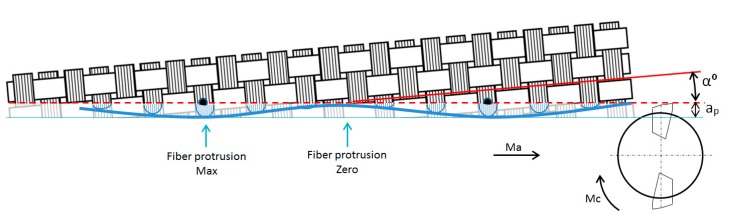
Sine wave effect.

Sine wave form is appreciated when machining direction is slightly inclined (α°) in respect to the fiber orientation (Figure 9), due to movement in the yarns during RTM process or tool position during machining.

**Figure 10:**
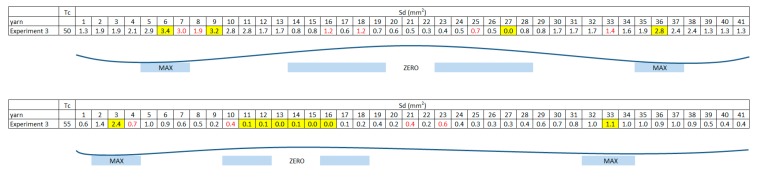
Sine wave effect.

Figure 10 shows values of Sd for every fill yarn, and the wave effect described previously can be appreciated. Red numbers show deformation in the plain weave and yellow maximums and minimums. Depending on the inclination of the cutting path to the yarn, the amplitude of the sine wave varies.

### 3.3. Definition of Sd/L Parameter to Compare Delamination Values

To compare delamination types among experiments a delamination parameter (Sd/L) is needed. In this parameter, a length (L) of 80 mm is evaluated, as well as the measured area of each type of delamination (as shown in Table 3 and Table 4). This parameter is similar to the one used to measure Ra in surface roughness. Table 5 shows Sd/L parameter, with cutting length (CL) and Xd measured.

Xd measurement is difficult. It can only be measured in the edges of the laminate (left Xd and right Xd). These values allow the calculation of the machining path of the tool on the yarn. Theoretical values of Xd did not coincide with observed values in every fill yarn along the length of the laminate, because plain weave form fibers move and deform in the mold during the RTM process because of injection pressure and plain weave manipulation. Due to this, expected area (Sd) value in Type II delamination did not coincide with the measured value.

**Figure 11:**
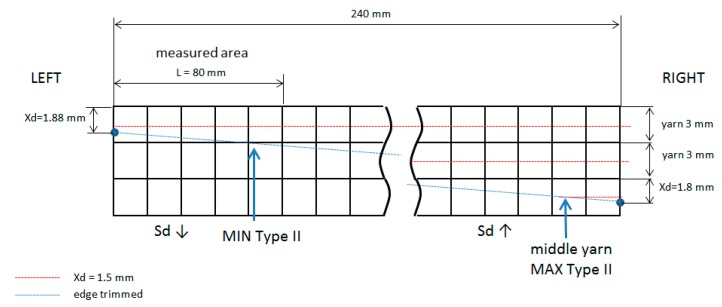
Xd values and cutting path in Test 1 at 30 min.

A qualitative trend can be observed, distinguished between, if the cutting tool path was inclined or not. If machining cutting (edge trimmed) advances to a yarn crossing, Sd decreases, and a value lower than expected is obtained (Sd ↓). If machining cutting path advances to a yarn center Sd increases, and a value higher than expected is obtained (Sd ↑). In every experiment, it can be explained why Sd is different than expected (Figure 11).

**Figure 12:**
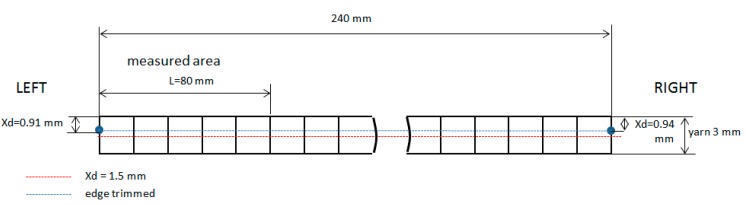
Xd values and cutting path in Test 1 at 55 min.

If the yarn direction and cutting path are coincident, Sd values in Type II of delamination are uniform (Figure 12) along the length, for instance Test 1 at 55 min, Test 3 at 30 min and Test 4 at 30 min.

**Figure 13:**
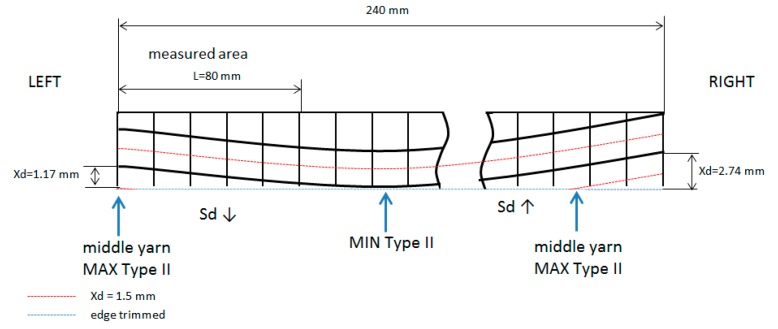
Xd values and cutting path in Test 1 at 80 min.

**Figure 14:**
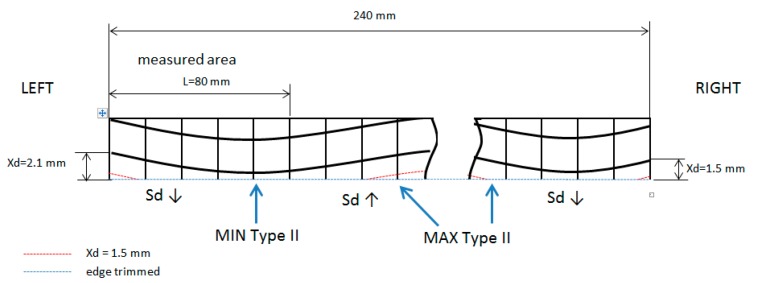
Xd values and cutting path in Test 3 at 80 min.

Figure 13 and Figure 14 show experiments to appreciate movements of the weave plain during resin transfer molding (RTM) process.

**Figure 15:**
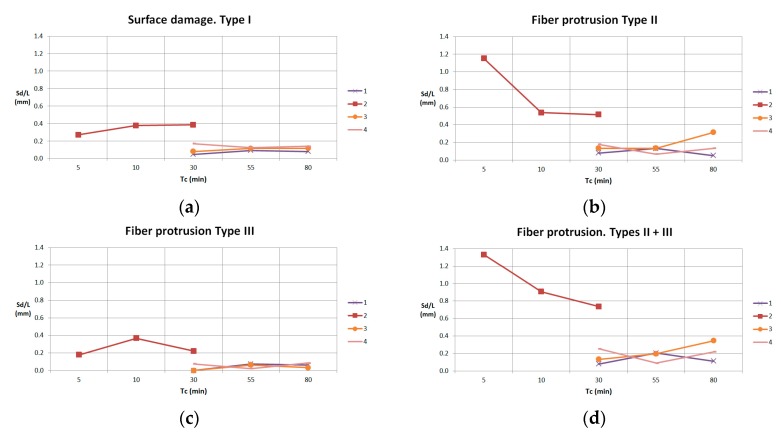
(**a**) Sd/L values for Type I delamination; (**b**) Sd/L values for Type II delamination; (**c**) Sd/L values for Type III delamination; (**d**) Sd/L values for Types II and III delamination.

In every experiment, parameter Sd/L is represented by each delamination type (Figure 15). Values of these parameters are similar in all experiments, except Experiment 2, because of the high flank wear of the tool.

**Figure 16:**
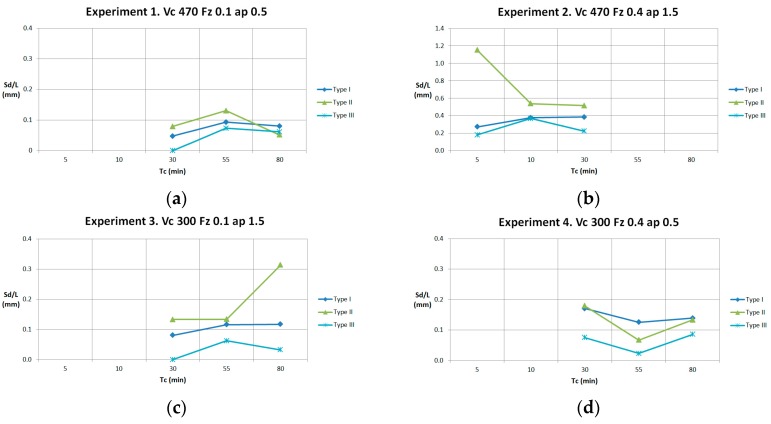
(**a**) Sd/L in Experiment 1; (**b**) Sd/L in Experiment 2; (**c**) Sd/L in Experiment 3; (**d**) Sd/L in Experiment 4.

Results of Sd/L, taking into account values of Xd, are shown in Figure 16. For instance, in Experiment 1 at 30 min and 80 min, trend for Sd is lower (Sd ↓) than the real value. For this reason, the points of the graph are not increasing.

### 3.4. Relation between Tool Wear and Delamination Parameters

**Figure 17:**
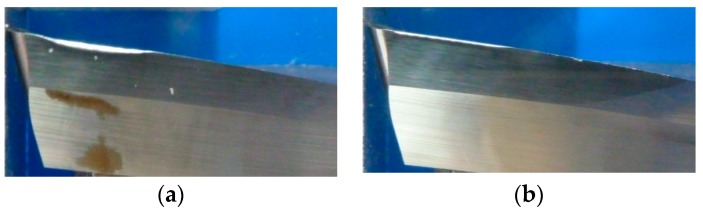
(**a**) Flank wear in Experiment 2; (**b**) Flank wear in Experiment 3.

High cutting conditions provokes high delamination and, in consequence, high tool wear, avoiding machining. In Figure 17, flank wear is shown at Experiments 2 and 3, with Vb values of 0.247 and 0.073 mm respectively.

**Figure 18:**
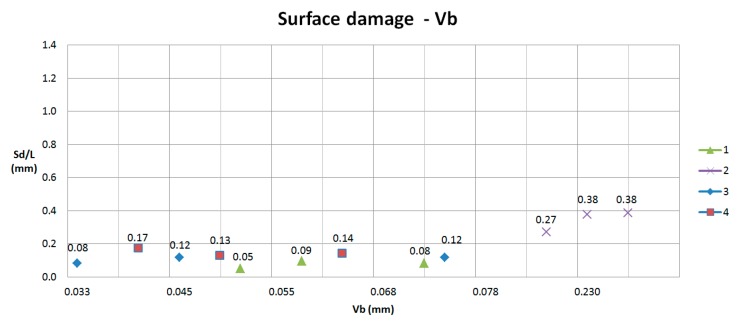
Sd/L respect Vb for surface damage (Type I).

**Figure 19:**
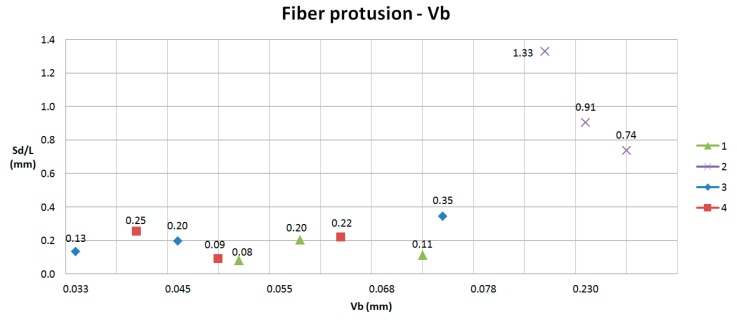
Sd/L respect Vb for fiber protrusion (Types II and III).

In Experiments 1, 3 and 4, after machining 80 min, flank wear does not reach the maximum tool wear to avoid machining, having a linear behavior and maintaining a low value (maximum flank wear tool is 0.073 mm), allowing machining until 240 m. For this reason, delamination is low and very similar in all experiments (Figure 18 and Figure 19).

## 4. Conclusions

This paper evaluates delamination in edge trimming of basalt fiber reinforced plastics (BFRP), manufactured by resin transfer molding (RTM), varying cutting conditions (cutting speed, feed rate and depth of cut) and fiber volume fraction. Cutting tool selection has been an important issue, allowing machining large cutting lengths and maintaining a low flank wear.

Delamination appears in the top layer of the laminate with a high fiber volume fraction (60%). If fiber volume is lower, resin layer at the top prevent delamination defect. In plain weave sample machining, relation between fiber orientation and position in the laminate to machining direction generate two different kinds of delamination: uniform and sine wave form.

Three types of delamination (I, II and III) have been evaluated, increasing their values as cutting speed and feed rate increases. Delamination values are maintaining in low values, allowing cutting lengths until 240 m, except with high cutting conditions. In this case, cutting is not possible for a high tool wear, which provokes high delamination.

Delamination prediction is not an easy task, because of its random nature. This is due to the movement of fibers during the RTM process and the position of the trimmed edge to the yarns. To explain delamination values of each type, a parameter relating surface areas and length (Sd/L) has been evaluated, together with the distance of the warp yarn from the trimmed edge until the next dip below the crossing fill yarn (Xd).

Type II delamination is the most usual. In Type II delamination the maximum value of fiber protrusion is higher than depth of cut, because originally the fill yarn has a sine wave form and after cutting the yarn is stretched. For this reason, depth of cut is an important variable in delamination values.

## Figures and Tables

**Table 1:** Variables and levels.

Level	Cutting Speed Vc (m/min)	Feed per Tooth Fz (mm)	Depth of Cut ap (mm)	Fiber Volume Fv (%)
1	300	0.1	0.5	40
2	470	0.4	1.5	60

**Table 2:** Experimental parameters.

Test	Cutting Speed Vc (m/min)	Feed per Tooth Fz (mm)	Depth of Cut ap (mm)	Fiber Volume Fv (%)
1	470	0.1	0.5	60
2	470	0.4	1.5	60
3	300	0.1	1.5	60
4	300	0.4	0.5	60
5	470	0.1	1.5	40
6	470	0.4	0.5	40
7	300	0.1	0.5	40
8	300	0.4	1.5	40

**Table 3:** Surface damage values (Type I).

Test	Vc	Fz	ap	Fv %	Tc (min)	Vb (mm)	Type I		
							Sd (mm^2^)	% L	Vmax (mm)
1	470	0.1	0.5	60	30	0.049	3.80	41%	0.30
					55	0.057	7.48	69%	0.61
					80	0.072	6.43	58%	0.74
2	470	0.4	1.5	60	5	0.211	21.68	95%	0.96
					10	0.232	30.11	99%	2.29
					30	0.247	30.79	94%	2.28
3	300	0.1	1.5	60	30	0.032	6.46	45%	0.68
					55	0.047	9.26	73%	0.81
					80	0.073	9.36	95%	0.49
4	300	0.4	0.5	60	30	0.041	13.65	65%	1.83
					55	0.048	10.03	53%	1.43
					80	0.063	11.14	81%	1.14

**Table 4:** Fiber protrusion values (Types II and III).

Test	Vc	Fz	ap	Fv %	Tc (min)	Vb (mm)	Type II			Type III		Total	II and III
							Sd (mm^2^)	% L	Vmax	Sd (mm^2^)	% L	Sd (mm^2^)	% L
1	470	0.1	0.5	60	30	0.049	6.35	41%	0.614	0	0%	6.35	41%
					55	0.057	10.44	42%	0.611	5.89	43%	16.33	85%
					80	0.072	4.12	35%	0.655	4.94	38%	9.06	73%
2	470	0.4	1.5	60	5	0.211	92.17	48%	2.110	14.29	52%	106.46	100%
					10	0.232	43.04	45%	2.584	29.41	54%	72.45	99%
					30	0.247	41.21	39%	2.468	17.77	55%	58.98	94%
3	300	0.1	1.5	60	30	0.032	10.64	72%	1.487	0	0%	10.64	72%
					55	0.047	10.66	70%	0.993	5.00	22%	15.66	92%
					80	0.073	25.07	68%	2.104	2.59	30%	27.66	98%
4	300	0.4	0.5	60	30	0.041	14.34	55%	1.201	6.01	20%	20.35	75%
					55	0.048	5.35	40%	0.715	1.84	16%	7.19	56%
					80	0.063	10.66	55%	0.634	6.85	32%	17.51	87%

**Table 5:** Sd/L parameter.

Test	Vc	Fz	ap	Tc	Vb	CL (m)	Sd/L (mm)				Xd (mm)		Trend Type II
							TypeI	II	III	II + III	Left	Right	
1	470	0.1	0.5	30	0.049	35.9	0.05	0.08	0.00	0.08	1.88	1.8	Sd ↓
				55	0.057	65.8	0.09	0.13	0.07	0.20	0.91	0.94	uniform
				80	0.072	95.7	0.08	0.05	0.06	0.11	1.17	2.74	Sd ↓
2	470	0.4	1.5	5	0.211	23.9	0.27	1.15	0.18	1.33	-	-	-
				10	0.232	47.9	0.38	0.54	0.37	0.91	-	-	-
				30	0.247	143.6	0.38	0.52	0.22	0.74	-	-	-
3	300	0.1	1.5	30	0.032	22.9	0.08	0.13	0.00	0.13	0.9	0.95	uniform
				55	0.047	42.0	0.12	0.13	0.06	0.20	1.1	1.16	Sd ↓
				80	0.073	61.1	0.12	0.31	0.03	0.35	2.1	1.5	Sd ↑
4	300	0.4	0.5	30	0.041	91.7	0.17	0.18	0.08	0.25	2.11	2.3	uniform
				55	0.048	168.1	0.13	0.07	0.02	0.09	2.35	1.57	Sd ↓
				80	0.063	244.5	0.14	0.13	0.09	0.22	2.71	1.93	Sd ↓

## References

[1] Teti R. (2002). Machining of composite materials. CIRP Ann. Manuf. Technol..

[2] Voss R., Seeholzer L., Kuster F., Wegener K. (2017). Influence of fibre orientation, tool geometry and process parameters on surface quality in milling of CFRP. CIRP J. Manuf. Sci. Technol..

[3] Lopresto V., Caggiano A., Teti R. (2016). High Performance Cutting of Fiber Reinforced Plastic Composite Materials. Proc. CIRP.

[4] König W., Grass P. (1989). Quality definition and assessment in drilling of fibre reinforced thermosets. CIRP Ann. Manuf. Technol..

[5] Colligan K., Ramulu M. (1992). The effect of edge trimming on composite surface plies. Manuf. Rev. (USA).

[6] Azmi A.I., Lin R.J.T., Bhattacharyya D. (2013). Machinability study of glass fibre-reinforced polymer composites during end milling. Int. J. Adv. Manuf. Technol..

[7] Davim J.P., Reis P. (2005). Damage and dimensional precision on milling carbon fiber-reinforced plastic using design experiments. J. Mater. Process. Technol..

[8] Davim J.P., Campos Rubio J., Abrao A. (2007). A novel approach based on digital image analysis to evaluate the delamination factor after drilling composite laminate. Compos. Sci. Technol..

[9] Gaitonde V.N., Karnik S.R., Campos Rubio J., Esteves Correia A., Abrão A.M., Davime J.P. (2008). Analysis of parametric influence on delamination in high-speed drilling of carbon fiber reinforced plastic composites. J. Mater. Process. Technol..

[10] Hintze W., Hartmann D., Schütte C. (2011). Occurrence and propagation of delamination during the machining of carbon fibre reinforced plastics (CFRPs)–An experimental study. Compos. Sci. Technol..

[11] Hintze W., Cordes M., Koerkel G. (2015). Influence of wear structure on delamination when milling CFRP. J. Mater. Process. Technol..

[12] Razfar M.R., Zanjani Zadeh M.R. (2009). Optimum damage and surface roughness prediction in end milling glass fibre-reinforced plastics, using neural network and genetic algorithm. Proc. Inst. Mech. Eng. Part B J. Eng. Manuf..

[13] Sheikh-Ahmad J.Y., Dhuttargaon M., Cheraghi H. (2017). New tool life criterion for delamination free milling of CFRP. Int. J. Adv. Manuf. Technol..

[14] Sheikh-Ahmad J.Y., Urban N., Cheraghi H. (2012). Machining Damage in Edge Trimming of CFRP. Mater. Manuf. Process..

[15] Navarro M.D., Meseguer M.D., Sánchez A.I., Gutiérrez S.C. (2017). Tool wear study in edge trimming on basalt fibres reinforced plastics. Proced. Manuf..

[16] Lopresto V., Leone C., De Iorio I. (2011). Mechanical characterisation of basalt fibre reinforced plastic. Compos. Part B.

[17] Fiore V., Scalici T., Di Bella G., Valenza A. (2015). A review on basalt fibre and its composites. Compos. Part B.

[18] Fiore V., Di Bella G., Valenza A. (2011). Glass-basalt/epoxy hybrid composites for marine applications. Mater. Des..

